# Metagenomics Reveals the Diversity and Taxonomy of Carbohydrate-Active Enzymes and Antibiotic Resistance Genes in Suancai Bacterial Communities

**DOI:** 10.3390/genes13050773

**Published:** 2022-04-27

**Authors:** Qiaozhi Song, Binbin Wang, Ye Han, Zhijiang Zhou

**Affiliations:** School of Chemical Engineering and Technology, Tianjin University, Tianjin 300072, China; songqiaozhi95@tju.edu.cn (Q.S.); binbinxin100@163.com (B.W.); hanye@tju.edu.cn (Y.H.)

**Keywords:** Chinese northeast suancai, metagenome, CAZymes, antimicrobial resistance genes, bacteria, food safety

## Abstract

Suancai, as a traditional fermented food in China with reputed health benefits, has piqued global attention for many years. In some circumstances, the microbial-driven fermentation may confer health (e.g., probiotics) or harm (e.g., antibiotic resistance genes) to the consumers. To better utilize beneficial traits, a deeper comprehension of the composition and functionality of the bacterial species harboring enzymes of catalytically active is required. On the other hand, ingestion of fermented food increases the likelihood of microbial antibiotic resistance genes (ARGs) spreading in the human gastrointestinal tract. Besides, the diversity and taxonomic origin of ARGs in suancai are little known. In our study, a metagenomic approach was employed to investigate distribution structures of CAZymes and ARGs in main bacterial species in suancai. Functional annotation using the CAZy database identified a total of 8796 CAZymes in metagenomic data. A total of 83 ARGs were detected against the CARD database. The most predominant ARG category is multidrug-resistant genes. The ARGs of antibiotic efflux mechanism are mostly in Proteobacteria. The resistance mechanism of ARGs in Firmicutes is primarily antibiotic inactivation, followed by antibiotic efflux. Due to the abundance of species with different ARGs, strict quality control including microbial species, particularly those with lots of ARGs, is vital for decreasing the risk of ARG absorption via consumption. Ultimately, we significantly widen the understanding of suancai microbiomes by using metagenomic sequencing to offer comprehensive information on the microbial functional potential (including CAZymes and ARGs content) of household suancai.

## 1. Introduction

The consumption of traditional fermented food is very widespread, with renowned health benefits [1]. The metabolic activities of microbiota cause fermentation, which converts natural ingredients in food into a diverse range of molecules that constitute the unique composition of the eventual fermented food [2]. The microbial diversity is unique to each food type and influenced by the ingredients in the manufacturing process [3]. In homemade fermented food, various microorganisms that contribute to traditional fermentation come mostly from the environment, and especially from raw materials of fermented food [4]. Suancai is a traditional fermented food depending on traditional approaches in the northeast of China, where it is one of the most significant fundamental foodstuffs. During the preparation of traditional Chinese suancai, spontaneous fermentation without the use of starter cultures or sterilization results in the proliferation of numerous microorganisms. On account of the crucial role of the microorganisms in the fermentation process, a thorough comprehension of the functional potential of the suancai microbiota is essential for improving the flavor and safety of traditional fermented food.

Metagenomic sequencing is shown to be an effective approach for defining the microbiota in fermented foods, obtaining species-level taxonomic resolution and predicting the functional potential [5,6]. Metagenome sequencing aids in the study of biocatalysts biodiversity in nature. That is to say, metagenomics has propelled synthetic biology study forward by discovering expression systems, proteins and bioactive compounds with a wide range of industrial applications [7,8]. It is desirable to investigate traditional homemade suancai for uncovering the microbial communities harboring biotechnologically important enzymes that are catalytically active during fermentation.

Due to the misuse or overuse of antibiotics in agricultural, animal husbandry and human medical situations, ARGs have received a lot of attention around the world as an emergent environmental genetic contaminant [9,10,11]. ARGs have been found in a large number of microbial genomes [12]. Concerns about ARGs in fermented products should be a priority, given the possibility that certain microbes could shape the gut microbiome via fermented food supplements [13,14]. That would mean that food (meat and vegetables) not only acts as a reservoir for ARGs and antibiotic resistance (AR) bacteria, but also as a mediator for the transmission of ARGs and AR bacteria from the surroundings to humans via food consumption [15,16]. As a result, it is critical to improve our understanding of the existence and transmission of ARGs through food consumption [17]. A variety of ARGs encoding resistance to a wide range of antibiotics have been discovered in foodborne bacteria [18,19]. Many species-centric studies focused on the relationships between AR bacteria and the ARGs they hold [20]. The findings revealed that species belonging to the genera *Enterococcus*, *Lactobacillus*, *Streptococcus*, *Lactococcus*, *Pediococcus* and *Weissella* harbor genes conferring resistance to tetracycline, vancomycin, macrolide, erythromycin and streptomycin [21,22,23]. The ARGs of AR bacteria could be transmitted to bacteria in the gastrointestinal system through horizontal gene transfer, so it is critical to reduce the spread of AR through consumption.

Current research on foodborne bacteria primarily focuses on individual pathogens and identifier microbes [24]. ARGs allow bacteria to survive throughout the face of antibiotics, and the resistance to antibiotics is reliably evaluated by phenotypic testing of isolates to a variety of antibiotics in food microbiology labs [25]. Nevertheles, the time required for this method, which relies on bacterial growth rates, can vary from one day to several weeks, and a high proportion of microbiota cannot be isolated in standard culture media [26,27]. Furthermore, horizontal gene transfer (HGT) mechanisms have shown that commensal and beneficial bacteria can acquire antibiotic resistance from pathogenic strains, highlighting the importance of studying the entire ARGs from the entire bacterial community (resistome) rather than single isolates [28,29]. Food microbiology is being revolutionized by metagenomics, which has resulted in a huge change from phenotype-based to genotype-based antibiotic resistance identification [30]. Nevertheless, the ARGs distribution may not fully represent the actual antibiotic resistance phenotypes of the microbial taxa, especially in the case of dead bacteria [31]. Nonetheless, ARG profiles do reveal the resistance potentials of microbial species in varying circumstances and with various antibiotic types. More importantly, a high throughput metagenomic approach can comprehensively provide insights into the complex community of microbial species (microbiome) as well as the pattern of antibiotic resistomes carried by those species [24,32].

In this study, three samples were collected at different stages in the suancai fermentation process, and the distribution and phylogenetic patterns of carbohydrate-active enzymes and ARGs were determined by a metagenomic approach. Our research will provide a foundation for future function mining of suancai microbiome.

## 2. Materials and Methods

### 2.1. Sample Collection and Sequencing

In this study, Chinese northeast suancai was processed and samples were collected at different time points following the procedure we previously described [33]. Briefly, the suancai brine was thoroughly mixed before being collected from the upper, middle and lower layers of the jar respectively. The samples were collected every day during the fermentation process for physicochemical index measurement in triplicates. The nitrite content showed an increasing trend at the beginning of fermentation (before day 3), which accumulated a nitrite peak at day 3. Afterwards the nitrite content sharply decreased, finally reaching a stable value at day 7. Based on the nitrite concentration, samples A (day 3), B (day 5) and C (day 7) during the fermentation were selected for sequencing. Metagenomic DNA was extracted using the QIAamp DNA Microbiome kit following the manufacturer’s protocol (QIAGEN Inc., Germany). Sequencing libraries were generated from metagenomic DNA (1 μg) using NEBNext^®^ Ultra™ DNA Library Prep Kit for Illumina (NEB, USA) according to the manufacturer’s protocol. Index codes were added to attribute sequences to each sample. In brief, DNA libraries of fragments (size of 350 bp) were prepared respectively for each sample. The samples were sequenced on the Illumina NovaSeq 6000 platform at Novogene Bioinformatics Technology Co., Ltd. (Tianjin, China).

### 2.2. Metagenome Assembly and Taxonomic Assignment

Raw data was preprocessed in order to obtain clean data for subsequent analysis. The detailed processing steps for quality control are provided in the Supporting Information. The clean data were assembled and analyzed by SOAP denovo software V2.04 [34]. The assembled scaftigs were then disconnected from N connection, leaving the Scaftigs without N. The samples’ clean data were mapped to each scaffold separately by Bowtie software V2.2.4 to obtain the reads that were not used, which were then combined and processed as mentioned above for mixed assembly. Using the number of reads and the length of the genes on alignment, the abundance of each gene in each sample was calculated. The equation was shown as follows, *r* represents the number of reads matched to the genes and *L* represents the length of genes [35,36,37].

$$G_{k}=\frac{r_{k}}{L_{k}}\cdot \frac{1}{\sum_{i=1}^{n}\frac{r_{i}}{L_{i}}}$$

To obtain the taxonomic annotation, the amino acid sequences of the predicted genes were aligned in the NCBI nr database with DIAMOND (blastp, cut-off *E*-value of 1 × 10^−5^) [38]. Taxonomic abundances were normalized by dividing the number of reads of a specific taxon by the total number of reads assigned to bacterial 16s rRNA in the sample.

### 2.3. Functional Annotation

To gain knowledge of the main functional and metabolic pathway, Kyoto Encyclopedia of Genes and Genomes (KEGG) [39,40], Evolutionary Genealogy of Genes: Nonsupervised Orthologous Groups (eggNOG) [41] and Carbohydrate-Active enzymes (CAZy) [42] databases were used for functional annotation of genes. Unigenes were aligned against these databases by using BLASTP, the mapped contigs were screened with an e-value threshold of 1 × 10^−5^. In the case of each sequence’s blast result, the best blast hit was used for further analysis [43,44].

### 2.4. ARGs Identification

Antimicrobial resistome analysis was carried out by aligning unigenes to CARD database v2.0.1 [45] using the blastp, e-value ≤ 1 × 10^−30^. The ARG abundance was expressed as fragments per kilobase per million fragments of contigs containing ARGs. Based on the aligned result by the Resistance Gene Identifier (RGI) tool, the abundance distribution of resistance genes in each sample, the taxonomic attribution analysis and the resistance mechanism of ARGs analysis were performed.

### 2.5. Statistical Analysis

R-3.5.1 was used for statistical analysis. The heatmaps were transformed into Z values on the base of relative abundance and were performed with “pheatmap” packages. A dissimilarity matrix was generated on the basis of the abundance of unigenes using the Bray–Curtis index [46] with package vegan. To identify the number of shared ARG subtypes across three samples, a Venn diagram was created by jvenn (a Venn tool).

## 3. Results and Discussion

### 3.1. Metagenomic Assembly Revealed CAZymes

**Figure 1:**
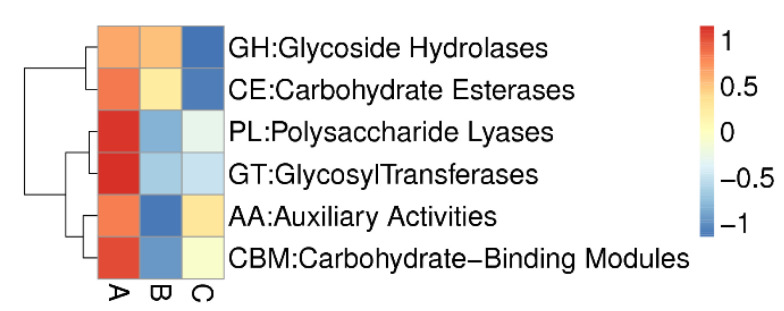
Comparative CAZy families heatmap of the suancai samples at three time points.

**Figure 2:**
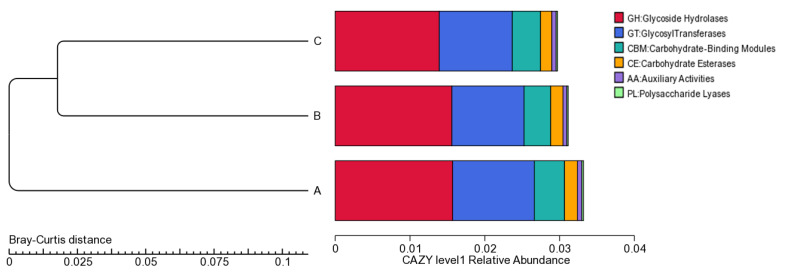
Composition of CAZyme-encoding genes. The cluster tree is based on the Bray–Curtis distance of CAZy relative abundance.

**Figure 3:**
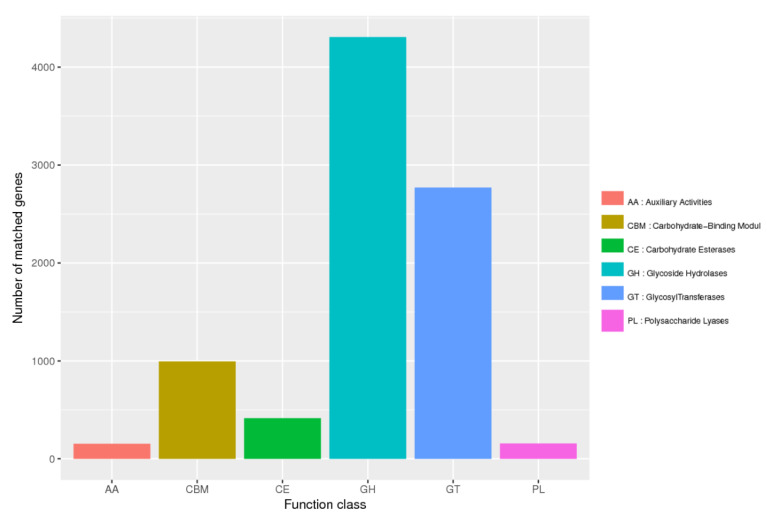
Metagenomic contigs mapped to CAZy family genes.

Both eggNOG-based and KEGG-based results revealed the richness of functional capabilities in relation to carbohydrate transport and metabolism and amino acid metabolism in the suancai metagenomic data (Figure S1). Functional domains for synthesis, degradation and modification of complex carbohydrates are regarded as CAZymes (Carbohydrate-Active enzymes). The CAZy database is used to annotate CAZyme-encoding genes belonging to the six CAZy families: glycoside hydrolases (GHs), glycosyltransferases (GTs), polysaccharide lyases (PLs), carbohydrate esterases (CEs), auxiliary activities (AAs) and carbohydrate-binding modules (CBMs). The metagenomic contigs of the suancai samples were queried against the CAZy database, which revealed the highest number of CAZyme-encoding genes in sample A at each family (Figure 1). In line with the Bray–Curtis distance based on CAZy relative abundance, the CAZyme-encoding genes belonging to the six CAZy families are closer between B and C (Figure 2), which is in agreement with the eggNOG and KEGG analyses (Figure S2). This reflects that the changes of microbiota composition cause different genes functioning at varying time points in suancai fermentation. A total of 8796 putative CAZymes were discovered in the metagenomic results (Figure 3). To be specific, the maximum number of contigs were matched to GHs (4306), followed by GTs (2770) across the three metagenomes. The remaining putative CAZyme hits were assigned to CBMs (994), CEs (415), PLs (157) and AAs (154). GTs, at high percentages in the metagenomic data, are acknowledged to catalyze the glycosidic linkages synthesis by transferring sugar moiety from phospho-activated sugar donors to saccharide or non-saccharide acceptors. The biosynthesis of disaccharides, polysaccharides and oligosaccharides is conductd by glycosyltransferase reactions [47].

### 3.2. Phylogenetic Distribution of CAZymes

**Figure 4:**
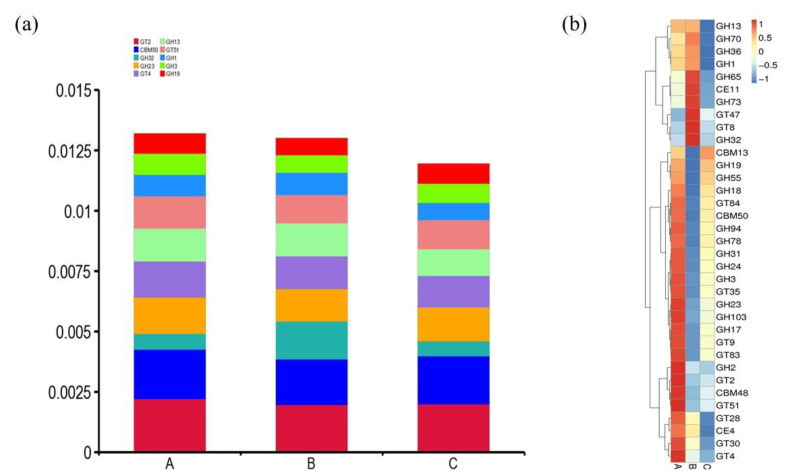
Comparative CAZymes analysis of metagenomic data sets of fermented suancai at different time points. (**a**) Percentage of top 10 CAZymes. (**b**) Relative abundance variations of CAZymes represented by a heatmap.

Despite CAZymes being distributed throughout the suancai microbiome, phylogenetic results of CAZyme encoding contigs demonstrated that a substantial proportion of CAZymes was contributed by bacteria belonging to order *Pseudomonadales*, *Enterobacterales*, *Lactobacillales* and *Sphingobacteriales*. The top 10 CAZymes in our metagenomic data are shown in Figure 4a. CBMs, with carbohydrate-binding activity, enhance the catalytic functions of CAZymes via making the carbohydrate-active modules more accessible to target substrates [48]. The CBM50 family, which comprises various enzymes belonging to the GH18, GH19, GH23, GH24, GH25 and GH73 families, i.e., enzymes that cleave peptidoglycan or chitin, was most abundantly present among CBM modules. The presence of CBMs involved in binding to polysaccharides suggested efficient recognition of a wide spectrum of carbohydrate polymers by GH family enzymes.

Catabolic enzymes that catalyze the cleavage of O-glycosidic bonds in carbohydrates are known as Glycoside hydrolases (GHs). These are high-efficiency catalysts for hydrolysis of most dominant and prevalent carbohydrates. Metagenome sequences for encoding β-galactosidases (GH1), β-glucosidase (GH3), lytic transglycosylases (GH23) and other abundant enzymes were discovered. The heatmap depicted the variations in relative abundance of the top 35 CAZymes (Figure 4b). One of the dominant GH families was the GH13, which is subdivided into ~40 subfamilies. Among the key enzymes of the GH13 family are α-amylase, α-glucosidase, oligo-α-glucosidase, sucrose phosphorylase and branching enzyme.

GH70 enzymes are transglucosylases produced by lactic acid bacteria (LAB). Many LAB strains from fermented vegetables are considered to be potential probiotics with immunomodulatory activity in vitro and in vivo [49]. CAZymes in *Lactobacillus* were known to be important in probiotic function, biomass transformation and vegetable tissue softening. GH70 enzymes are very interesting biocatalysts with strong applications in the food, pharmaceutical and cosmetic sectors. Here we unveiled the microbiological distribution of GH family enzymes in suancai. Notably, GH70 enzymes were all mapped to *Leuconostoc*, belonging to species *L. mesenteroides*, *L. fallax*, *L. citreum*, *L. gelidum* and *L. carnosum* (File S1). Some of these species were reported to be able to produce large amounts of extracellular polysaccharides, which can be employed as prebiotics or for other purposes in the food industry [50]. The aforementioned species were also frequently found in fermented vegetables. As a result, we assumed that these predominant microbial LAB species might play vital roles in determining the functional and sensorial properties of suancai products. This provides a reference for the identification and characterization of GH70 enzymes in LAB.

### 3.3. Occurrence and Characteristics of ARGs during Suancai Fermentation

**Figure 5:**
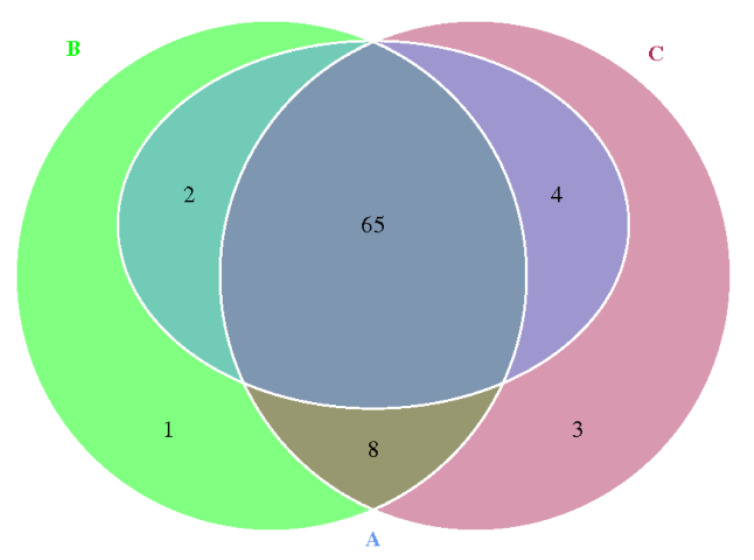
Venn diagram showing the number of shared and unique ARG subtypes among fermented suancai samples.

**Figure 6:**
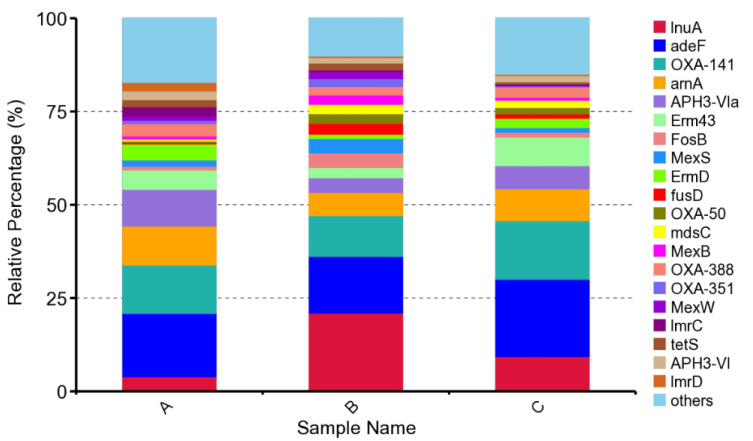
Composition and distribution of shared ARGs during suancai fermentation.

According to the results based on strict matches, the study characterized ARG occurrence and abundance in the suancai fermentation process. By using the CARD database and RGI tool, there were 65 shared ARGs among a total of 83 ARGs detected in three samples that were identifiable (Figure 5). Compared with sample B and C, sample A contained a lower diversity of resistance genes. The diversity abundance of ARGs increased obviously during suancai fermentation. The top 20 abundant ARGs accounted for over 80% of all the annotated ARGs and were considered to be representative ARGs (>80%) (Figure 6). This suggested that the distribution of ARGs was concentrated in suancai.

**Figure 7:**
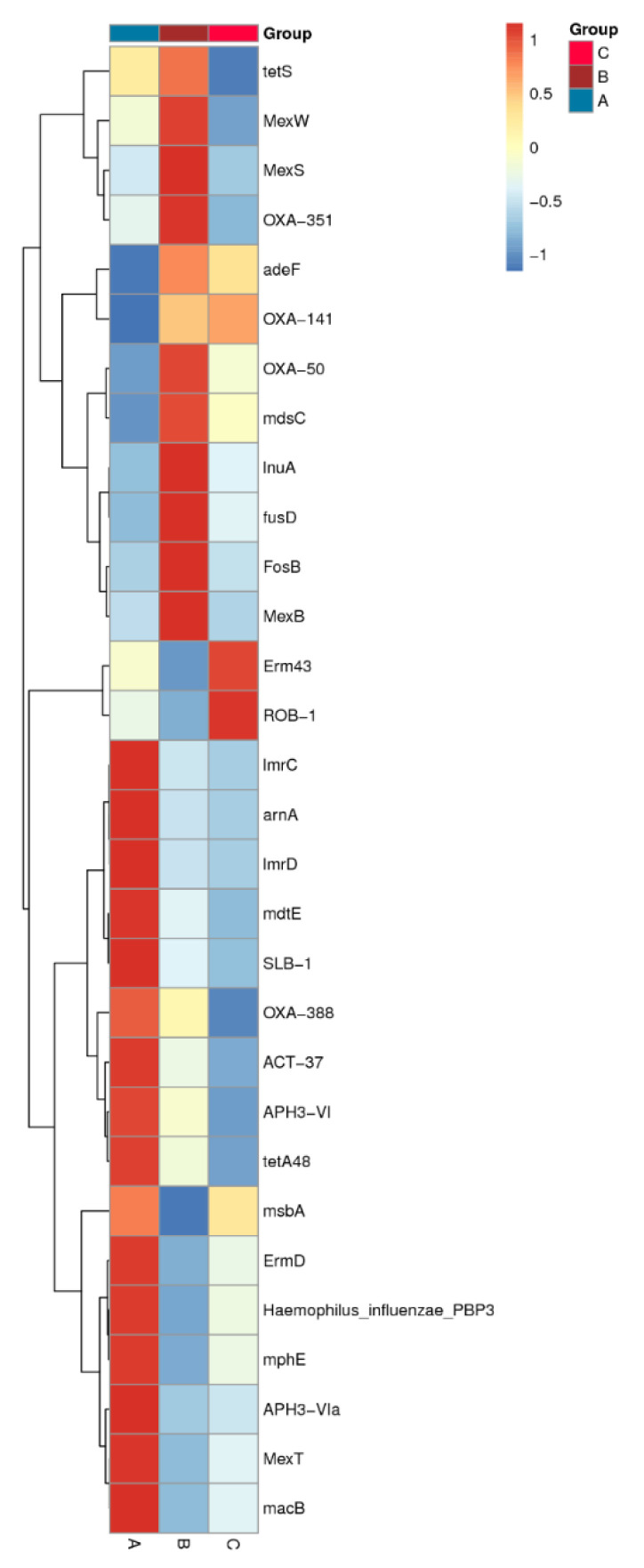
Heatmap of variations of ARGs based on relative abundance across three samples. The top 30 ARGs are shown. The right vertical axis is the ARG name, the clustering tree on the left vertical axis is the clustering tree of ARG, and the corresponding value of the heatmap is the Z value of the relative abundance of each row of ARG after normalized processing.

Using KEGG, the representative ARG subtypes identified mainly annotated to different classes (Table 1): multidrug resistance gene (*adeF*, *OXA-141*, *Erm43*, *MexS*, *ErmD*, *OXA-50*, *mdsC*, *MexB*, *OXA-388*, *OXA-351*, *MexW*), lincosamide (*lnuA*, *lmrC*, *lmrD),* aminoglycoside (*APH3-Vla*, *APH3-VI*), peptide (*arnA*), fosfomycin (*fosB*), fusidic acid (*fusD*) and tetracycline (*tetS*) resistance genes. Each sample has matched these representative resistance genes. Across three suancai samples, multidrug resistance was the most frequently assigned gene category. Microorganisms tend to develop multidrug resistance to counter environmental pressures. The multidrug resistance genes *adeF* and *OXA-141* are the prevalent ones in distribution; to be specific, the relative abundance in each sample exceeded 10% (Table S1). For sample B, the lincosamide antibiotic gene *lnuA* was the most abundant ARG: it was significantly increased compared to those in sample A or sample C. The genes discovered in suancai samples encoded resistance against lincosamides, aminoglycosides, macrolides, phenicols, fluoroquinolone and tetracyclines etc, whereas the ARGs of the product itself might reduce the efficacy of these antibiotics. Figure 7 demonstrates that the relative abundance of most ARGs become lower during fermentation, and are lowest in sample C. This phenomenon, together with no additives being used during traditional household suancai fermentation, suggests that the primary source of ARGs might mainly be a direct result of raw materials.

### 3.4. Correlation of ARGs and Their Potential Hosts

To confirm and compare the microbial origin of ARGs with the total microbial genes, the ARGs and total microbial genes were assigned to different taxa using resistance gene identifier (RGI) in CARD Resistance Database. The species attribution analysis of resistance genes was conducted (File S2). Taxonomic annotation revealed that most of the dominant species that matched with ARGs were assigned to *Pseudomonas* (*P. fluorescens, P. taetrolens* and *P. fragi*), *Serratia* (*Serratia* sp. *Leaf51*), *Erwinia* (*E. amylovora*, *E. pesicina*), *Stenotrophomonas* (*S. maltophilia*), *Rahnella* and some LABs, such as *Leuconostoc* (*L. gelidum*, *L. carnosum*), *Lactobacillus* (*Lactobacillus versmoldensis* and *Lactobacillus sakei*), *Lactococcus* (*Lactococcus lactis*) and *Weissella* (*W. soli*). The majority of the ARG-carrying species belonged to the *Pseudomonas* genus. They are common inhabitants in fermented vegetables due to the cold storage and their flexibility in nutritional requirements, which makes suancai a suitable substrate for them to grow.

**Figure 8:**
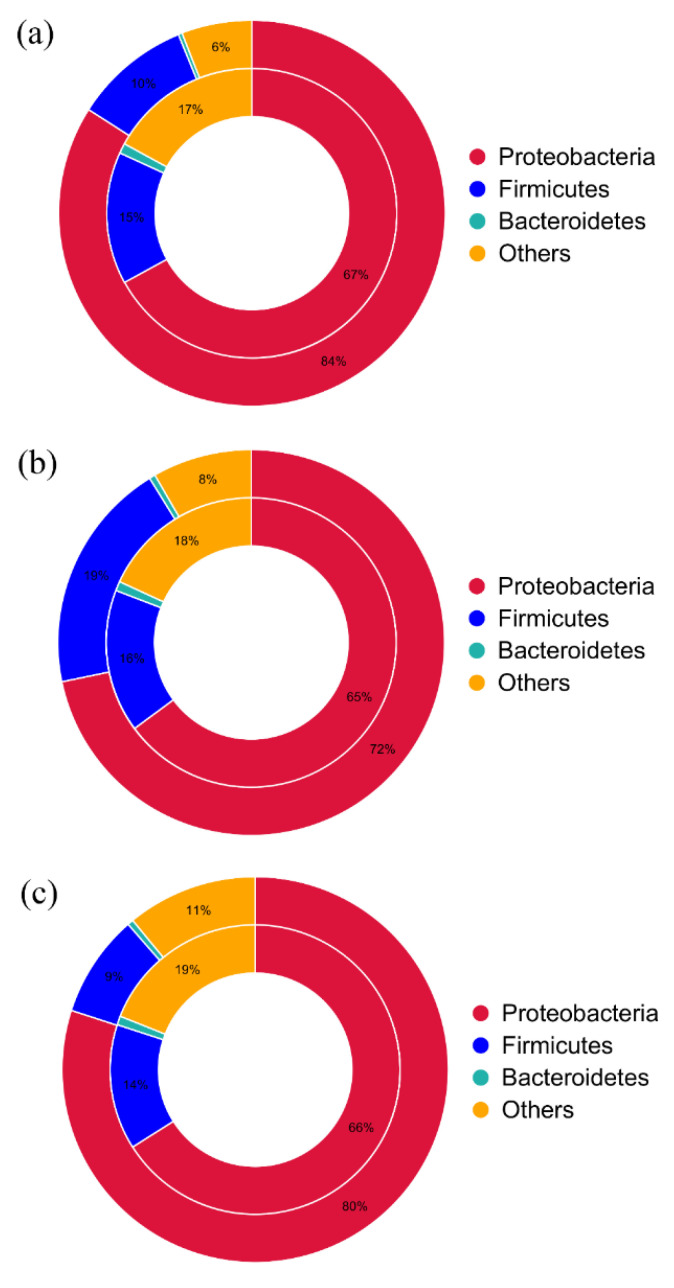
Comparison between the distribution of the ARGs (internal cycle) and the total microbial gene set (external cycle) at bacterial phylum level.

Figure 8 shows the taxonomic attribution results at phylum level. In sample A, the distribution of ARGs and total microbial genes at the phylum level was 67% and 84% for Proteobacteria, 15% and 10% for Firmicutes, respectively (Figure 8a). In sample B, the assignment of ARGs and total microbial genes at the phylum level was 65% and 72% for Proteobacteria, 16% and 19% for Firmicutes (Figure 8b). In sample C, the distribution of ARGs and total microbial genes at the phylum level was 66% and 80% for Proteobacteria and 14% and 9% for Firmicutes (Figure 8c). According to the findings, the majority of ARGs in homemade northeast suancai are found in Proteobacteria and Firmicutes. Somewhat differently, a previous study characterized the profiles of ARGs in ready-to-eat vegetables showed that the phylum-level assignment of ARGs and total microbial genes was 62% and 39% for Proteobacteria, 17% and 31% for Firmicutes respectively [14]. Its result showed that compared to other genes, ARGs were more likely to be found in Proteobacteria. However, in our homemade suancai, ARGs were more prone to exist in Firmicutes. The reason might be that most industrial ready-to-eat vegetable foods were produced by using starter cultures to initiate the fermentation, which probably contributes to their reduced diversity compared to spontaneous fermented foods [51]. This is unsurprising, given that homemade spontaneous fermented suancai has not been sterilized or treated with food additives that kill pathogenic as well as health-promoting/probiotic organisms. Furthermore, because homemade raw suancai is more vulnerable to environment and contamination during handling, its bacterial diversity is likely to be higher. This is in line with previous research which demonstrated ARGs varied across food substrate and between starter-type and spontaneous fermentations [51].

In our study, *APH3-Vla* and *APH3-VI* belonging to the APH gene family originated from Gammaproteobacteria (including Yersiniaceae and Pseudomonadales). The presence of *APH3-Vla* and *APH3-VI* in fermented suancai is consciously worrying as aminoglycoside 3′-phosphotransferases can mediate high-level resistance against a few aminoglycosides. These genes could be carried on plasmids or encoded on chromosomes; *APH3* is the latter, but a transposon-mediated mechanism for spreading resistance genes has been proposed [52,53]. Because the gene had previously only been described in *P. aeruginosa*, and was recently reported to have allegedly originated from *L. mesenteroides* in yogurt, the pathways of resistance gene transfer associated with this gene should be evaluated further. The result shows that the abundance of *APH3-Vla* and *APH3-VI* is highest in sample A. This phenomenon, together with no additives during traditional household suancai fermentation, raises the suspicion that the source of the *APH* may be a direct result of raw materials. With regard to the analytical data obtained in this study, some of the recognized ARG hosts were reported previously. For example, the resistance gene *MexVW* is commonly carried by *Pseudomonas* [54], and the resistance gene *emrD* has been determined in *Enterobacter* [55]. In our species attribution results, gene *ade*F, whose CARD ontology classifies it as a gene conferring resistance to tetracycline and fluoroquinolone antibiotics, is only attributed to phylum Proteobacteria (class Gammaproteobacteria). Gene *OXA-141*, as a broad spectrum β-lactamase previously detected in *P. aeruginosa*, is also only attributed to phylum Proteobacteria (class Gammaproteobacteria). Gene *lnuA*, a gene conferring resistance to lincomycin antibiotic, mapped to phylum Firmicutes (class Bacilli).

### 3.5. Resistance Mechanisms

**Figure 9:**
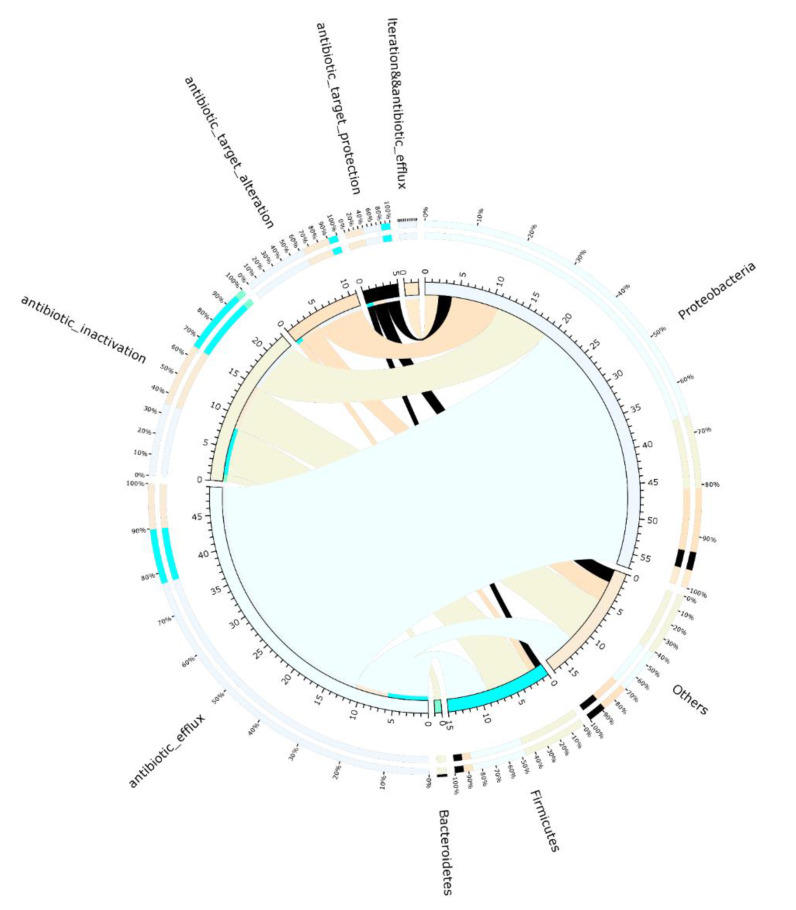
Distributions of ARG types in the microbial phyla. Details are shown in Table S2.

The percentage of resistance mechanisms was calculated for each sample based on the ARG abundances. In our suancai samples, the most dominant mechanism of detected ARGs was the antibiotic efflux, which included 36 genes, followed by antibiotic inactivation, which included 30 genes. The remaining resistance mechanisms, such as antibiotic target alteration and antibiotic target protection, only included 17 genes. Because results at lower taxonomic levels lack reliability, visual results for resistance mechanisms of microbiome are presented only at the phylum level (Figure 9). The result shows the ARGs of antibiotic efflux mechanism are mostly Proteobacteria. The resistance mechanisms of ARGs in Firmicutes are mostly antibiotic inactivation, followed by antibiotic efflux. Notably, the ARGs involve both antibiotic target alteration and antibiotic efflux mechanism found is only in *P. syringae* at the species level (Table S2), which is known as a plant pathogen. The ARGs mechanism of antibiotic target protection only involves tetracycline (*tetS*, *tetL*, *tet32*) resistance genes, and is only detectable in *Pseudomonas* and *Weissella* at genus level.

Compared with previous studies on kefir strains and yogurt products, the only mechanism discovered was antibiotic target protection. In one yogurt grain sample, antibiotic target alteration, antibiotic target replacement (51.28%) and antibiotic target protection (48.72%) are probable resistance mechanisms [56]. The mechanism differs markedly across fermented vegetables and dairy products. Microbial diversity and functional changes (e.g., AR) are driven by fermentation substrates in fermented foods, and the raw material has a significant influence on the resistance mechanisms of the microbiome in fermented foods. Compared to other fermented products, household fermented vegetables with more abundant microbes require more attention on resistance mechanisms of antibiotic efflux.

Metagenomics analyses in the study depend on shotgun DNA sequencing and cannot yet be directly linked to the phenotypes of antimicrobial resistance, especially those originating from dead bacteria. Nevertheless, published studies show that naturally competent bacteria can take up DNA released by dead microorganisms [57], implying their potential contribution to the transmission of ARGs. In terms of food security, quality control for microbial species with abundant and diverse ARGs is essential for minimizing the risk of ARGs incorporation during the consumption of traditional suancai. The findings of resistance mechanisms will serve as a guide for further control measures for specific microbial species.

## 4. Conclusions

In this study, a metagenome sequencing method was used to investigate the metagenomics of suancai, a traditional fermented food in the northeast of China. KEGG-based and eggNOG-based analysis results revealed a significant potential for carbohydrate transport and metabolism and amino acid metabolism. The species encoded kinds of CAZymes, notably GHs and GTs, implying their potential activities in carbohydrate metabolism. Phylogenetic analysis of CAZyme encoding contigs showed that a large proportion of CAZymes was contributed by bacteria belonging to order Pseudomonadales, Enterobacterales, Lactobacillales and Sphingobacteriales. GH70 enzymes were present in *L. mesenteroides*, *L. fallax*, *L. citreum*, *L. gelidum* and *L. carnosum*. Taken together, 8796 putative CAZymes were discovered in the metagenomic data providing a thorough understanding of the presence of diverse CAZymes in microbial species of suancai.

Although ARGs have been found in a variety of environments, little is known about their distribution and phylogenetic information in fermented foods. The alignment results against the CARD database showed the existence of *Pseudomonas* as the most abundant Gram-negative genus in fermented suancai bearing ARGs. Most ARGs exist in Proteobacteria and Firmicutes. The most predominant ARG category is multidrug-resistant genes. The four, mainly microbial, resistance mechanisms in suancai samples are antibiotic efflux, followed by antibiotic inactivation, antibiotic target alteration and antibiotic target protection. Therefore, it would be necessary to discreetly monitor the microbial subpopulation that holds ARGs and to optimize the sanitation conditions in suancai production processes to reduce the risk of drug-resistant genes transfer and develop effective strategies to control AR. This study revealed a wealth of information about carbohydrate-active enzymes and antibiotic resistance genes in suancai. The knowledge presented here will provide significant opportunities for improving suancai production and harnessing the health-promoting potential in the future.

## Figures and Tables

**Table 1:** Representative ARG subtypes and their abundance in each sample.

Items	ARG Type	Resistance Mechanism	Abundance in Sample A	Abundance in Sample B	Abundance in Sample C
lnuA	lincosamide	antibiotic inactivation×	1.53 × 10^−5^	0.000109	3.41 × 10^−5^
adeF	Multidrug	antibiotic efflux	6.55 × 10^−5^	7.86 × 10^−5^	7.56 × 10^−5^
OXA−141	Multidrug	antibiotic inactivation	5.00 × 10^−5^	5.67 × 10^−5^	5.74 × 10^−5^
arnA	peptide	antibiotic target alteration	4.03 × 10^−5^	3.20 × 10^−5^	3.12 × 10^−5^
APH3-VIa	aminoglycoside	antibiotic inactivation	3.80 × 10^−5^	2.05 × 10^−5^	2.26 × 10^−5^
Erm43	Multidrug	antibiotic target alteration	2.01 × 10^−5^	1.42 × 10^−5^	2.80 × 10^−5^
FosB	fosfomycin	antibiotic inactivation	3.83 × 10^−6^	2.02 × 10^−5^	4.69 × 10^−6^
MexS	Multidrug	antibiotic efflux	6.48 × 10^−6^	2.00 × 10^−5^	4.52 × 10^−6^
ErmD	Multidrug	antibiotic target alteration	1.66 × 10^−5^	6.00 × 10^−6^	9.15 × 10^−6^
fusD	fusidic acid	antibiotic inactivation	1.39 × 10^−6^	1.51 × 10^−5^	4.27 × 10^−6^
OXA-50	Multidrug	antibiotic inactivation	1.50 × 10^−6^	1.33 × 10^−5^	6.17 × 10^−6^
mdsC	Multidrug	antibiotic efflux	2.09 × 10^−6^	1.30 × 10^−5^	7.23 × 10^−6^
MexB	Multidrug	antibiotic efflux	3.21 × 10^−6^	1.29 × 10^−5^	2.87 × 10^−6^
OXA-388	Multidrug	antibiotic inactivation	1.28 × 10^−5^	1.16 × 10^−5^	1.00 × 10^−5^
OXA-351	Multidrug	antibiotic inactivation	3.55 × 10^−6^	1.11 × 10^−5^	9.50 × 10^−7^
MexW	Multidrug	resistance-nodulation-cell division (RND) antibiotic efflux pump	4.24 × 10^−6^	9.82 × 10^−6^	6.88 × 10^−07^
lmrC	lincosamide	antibiotic efflux	9.78 × 10^−6^	2.23 × 10^−6^	1.39 × 10^−6^
tetS	tetracycline	antibiotic target protection	7.24 × 10^−6^	9.75 × 10^−6^	1.94 × 10^−6^
APH3-VI	aminoglycoside	antibiotic inactivation	8.98 × 10^−6^	7.35 × 10^−6^	6.19 × 10^−6^
lmrD	lincosamide	antibiotic efflux	8.87 × 10^−6^	1.96 × 10^−6^	1.27 × 10^−6^

## Data Availability

The metagenomics sequences in this study were deposited in the SRA database in NCBI under the accession number SAMN16414836.

## Supplementary Materials

The following supporting information can be downloaded at: https://www.mdpi.com/article/10.3390/genes13050773/s1, Figure S1: Statistical map drawn from unigenes annotation results indicated substantial representation of carbohydrate metabolism in the metagenomes; Figure S2: The cluster tree of CAZy family genes based on Bray–Cutis distance; Table S1: Percentage abundance of annotated ARGs in three samples; Table S2: Profile of resistance mechanism and taxonomic distribution of ARGs. File S1: Taxonomic Attribution Information of All Annotated CAZymes in the Metagenomes. File S2: Taxonomic Attribution Information of All Annotated ARGs in the Metagenomes.
