# Supplementary material for: Metagenomics Reveals the Diversity and Taxonomy of Carbohydrate-Active Enzymes and Antibiotic Resistance Genes in Suancai Bacterial Communities
Source: Genes (Basel). 2022 Apr 27;13(5):773. doi: 10.3390/genes13050773 (PMC9141641; doi:10.3390/genes13050773)
Supplement: Supplementary file 1 [file genes-13-00773-s001.zip › genes-1662226-supplementary.pdf]

Supplementary Information

(a)

(b)

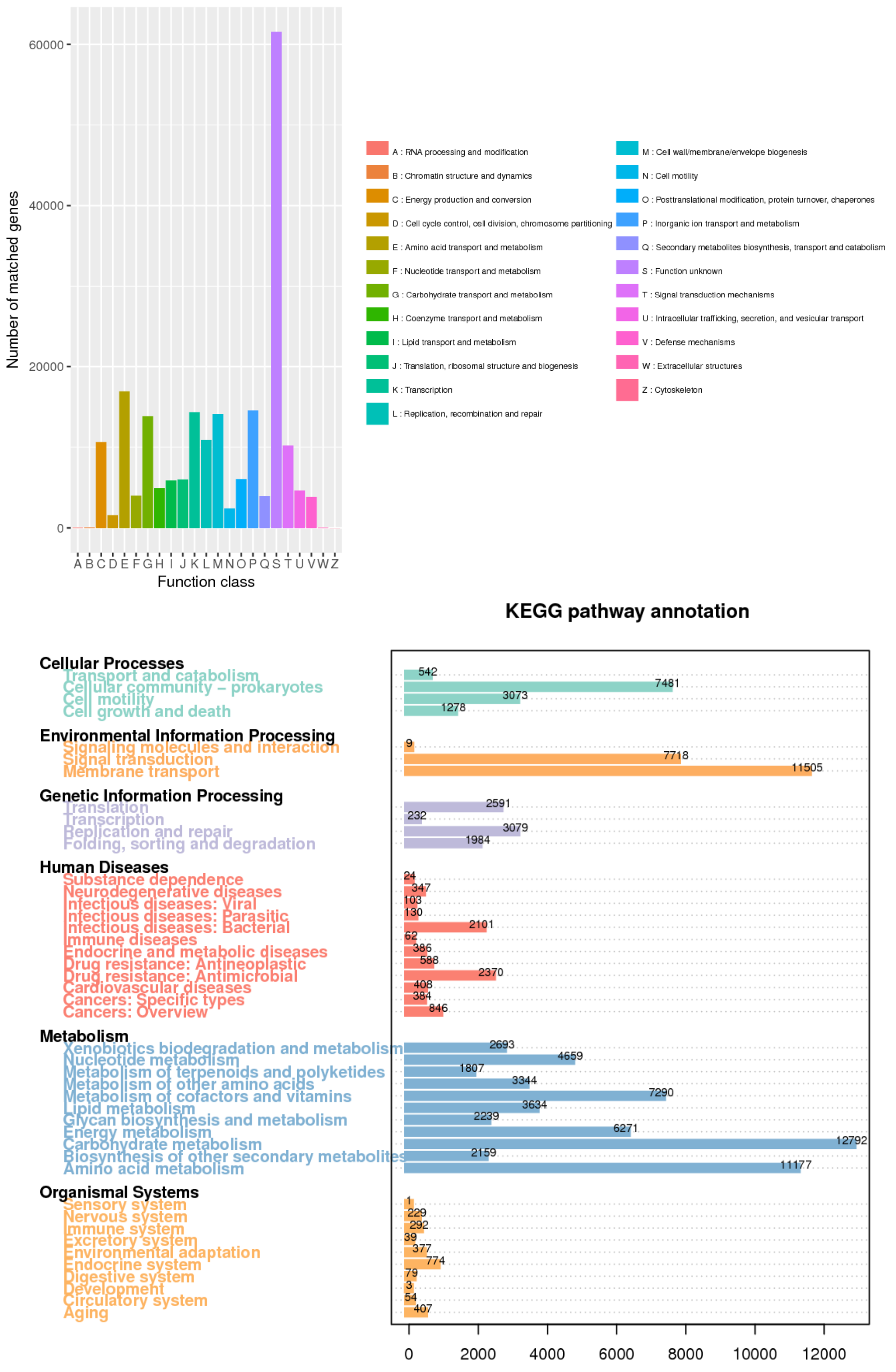

Figure S1. Statistical map drawn from unigenes annotation results indicated substantial representation of carbohydrate metabolism in the metagenomes. The number on the bar chart represents the number of annotated genes according to (a) eggNOG, (b) KEGG pathway annotation results, the other coordinate axis is the function classes in each database.

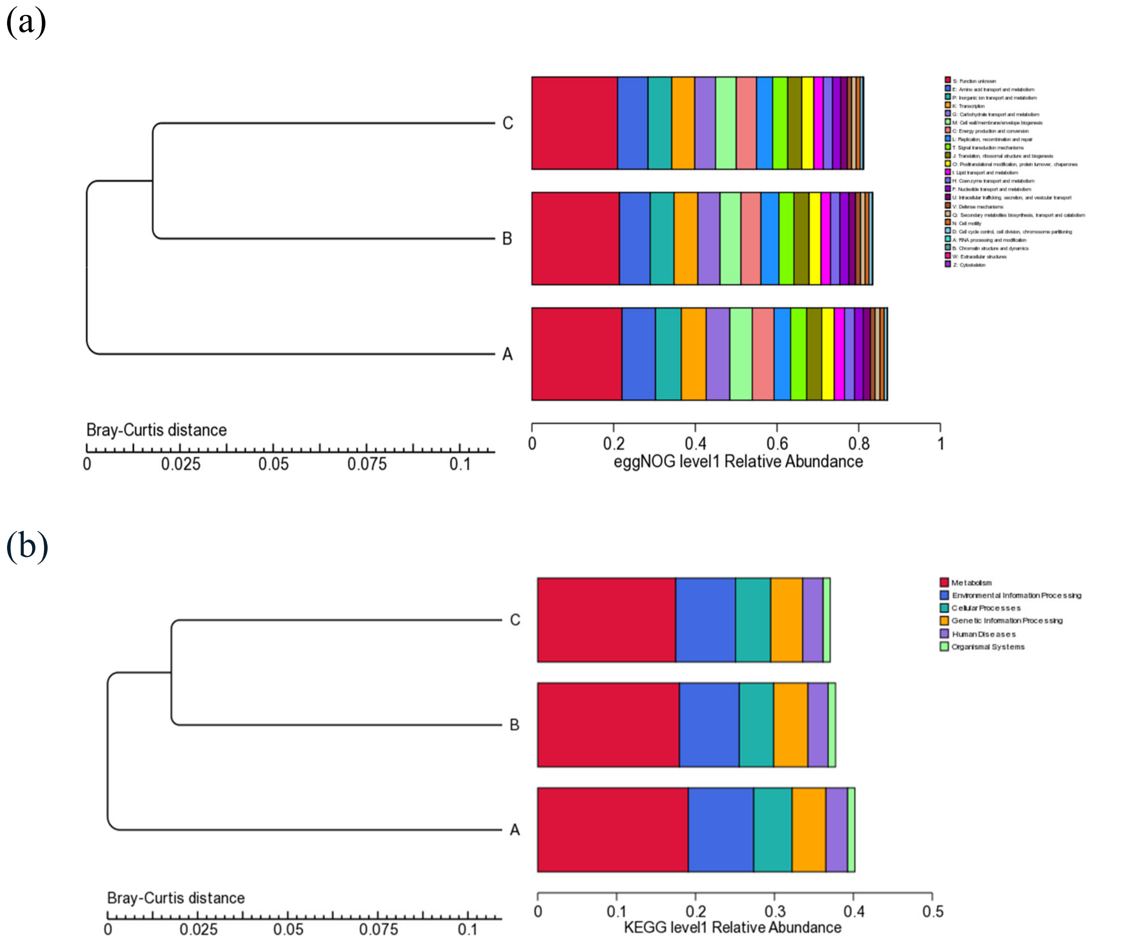

Figure S2. The cluster tree of CAZy family genes based on Bray-Cutis distance. The left is the structure of cluster tree, the right is the relative abundance distribution of of functional genes associated with (a) eggNOG (b) KEGG.

Methods

Quality control and metagenome assembly

The specific processing steps were as follows: (1) removal of the reads containing low-quality bases (quality threshold value ≤ 38) above 40 bp; (2) removal of the reads in which the N bases reached 10 bp; and (3) removal of the reads that shared an overlap of above 15 bp with the adapter. The clean data was assembled and analyzed with SOAP denovo software V2.04. Then, the assembled scaftigs were interrupted from N connection and leave the scaftigs without N. All samples' clean data were compared to each scaffolds respectively by Bowtie software V2.2.4 to acquire the reads not used, which were combined and then processed as described above for mixed assembly. The fragments shorter than 500 bp in all scaftigs were filtered out.

Table S1. Percentage abundance of annotated ARGs in three samples.

**Sample**	**A**	**B**	**C**
lnuA	3.97	21.01	9.34
adeF	16.96	15.18	20.71
OXA-141	12.94	10.95	15.72
arnA	10.45	6.19	8.55
APH3-VIa	9.85	3.96	6.19
Erm43	5.21	2.74	7.67
FosB	0.99	3.9	1.29
MexS	1.68	3.86	1.24
ErmD	4.29	1.16	2.51
fusD	0.36	2.92	1.17
OXA-50	0.39	2.57	1.69
mdsC	0.54	2.51	1.98
MexB	0.83	2.49	0.79
OXA-388	3.31	2.24	2.75
OXA-351	0.92	2.15	0.26
MexW	1.1	1.9	0.19
lmrC	2.53	0.43	0.38
tetS	1.87	1.88	0.53
APH3-VI	2.33	1.42	1.7
lmrD	2.3	0.38	0.35
others	17.19	10.15	15.01

Table S2. Profile of resistance mechanism and taxonomic distribution of ARGs.

**phylum**	**antibiotic _efflux**	**antibiotic in activation**	**antibiotic targ et alteration**	**antibiotic target alteration & ant ibiotic efflux**	**antibiotic target protection**
Others	5	7	3	0	2
Bacteroidetes	0	1	0	0	0
Firmicutes	6	7	1	0	1
Proteobacteria	38	8	7	2	2
**class**	**antibiotic efflux**	**antibiotic in activation**	**antibiotic targ et alteration**	**antibiotic target alteration & ant ibiotic efflux**	**antibiotic target protection**
Others	6	7	3	0	2
Flavobacteriia	0	1	0	0	0
Bacilli	6	7	1	0	1
Alphaproteobacteria	2	0	0	0	0
Betaproteobacteria	0	1	0	0	0
Gammaproteobacteria	35	7	7	2	2
**order**	**antibiotic efflux**	**antibiotic in activation**	**antibiotic target alteration**	**antibiotic target alteration & ant ibiotic efflux**	**antibiotic target protection**
Others	13	8	3	0	2
Flavobacteriales	0	1	0	0	0
Bacillales	0	0	1	0	0
Lactobacillales	6	6	0	0	1
Rhizobiales	2	0	0	0	0
Burkholderiales	0	1	0	0	0
Alteromonadales	1	0	0	0	0
Enterobacterales	11	1	0	0	0
Pseudomonadales	8	6	7	2	2
Xanthomonadales	8	0	0	0	0
**genus**	**antibiotic efflux**	**antibiotic inactivation**	**antibiotic target alteration**	**antibiotic target alteration & ant ibiotic efflux**	**antibiotic target protection**
Others	21	8	3	0	2
Chryseobacterium	0	1	0	0	0
Bacillus	0	0	1	0	0
Lactobacillus	1	1	0	0	0
Leuconostoc	3	4	0	0	0
Weissella	0	0	0	0	1
Lactococcus	2	1	0	0	0

Massilia	0	1	0	0	0
Shewanella	1	0	0	0	0
Erwinia	4	0	0	0	0
Rahnella	1	0	0	0	0
Serratia	2	1	0	0	0
Acinetobacter	1	0	1	0	0
Pseudomonas	6	6	6	2	2
Stenotrophomonas	7	0	0	0	0
**species**	**antibiotic efflux**	**antibiotic inactivation**	**antibiotic target alteration**	**antibiotic target alteration & antibiotic efflux**	**antibiotic target protection**
Others	33	14	10	1	3
Lactobacillus sakei	1	0	0	0	0
Lactobacillus versmoldensis	0	1	0	0	0
Leuconostoc carnosum	0	2	0	0	0
Leuconostoc gelidum	3	1	0	0	0
Weissella soli	0	0	0	0	1
Lactococcus lactis	2	1	0	0	0
Erwinia amylovora	1	0	0	0	0
Erwinia persicina	3	0	0	0	0
Serratia proteamaculans	1	0	0	0	0
Pseudomonas fluorescens	0	1	0	0	0
Pseudomonas fragi	0	2	0	0	0
Pseudomonas syringae	0	1	0	1	0
Pseudomonas taetrolens	0	0	1	0	1
Stenotrophomonas maltophilia	5	0	0	0	0
